# 

**Published:** 1845-10-01

**Authors:** 


*Buffalo City Association.*—Members will recollect that the regular monthly meeting of this Association will be on Tuesday the 7th inst., at 7 P. M., at the office of Dr. Bissell.

☞ For acknowledgments of publications &c., see cover—next page.

Buffalo Med. & Surg. Journ. Oct. 1845

### ACKNOWLEDGMENT OF PERIODICALS, &C.

We have received the following periodicals and other publications:

"*The field and the work of the Medical Profession.*" *An address delivered before the associated Alumni of Castleton, June 18th,* 1845, *by Horace Eaton, M. D.* An exceedingly well written discourse, abounding in just views and animating sentiments.—The writer is capable of doing signal service with his pen for any cause to which he chooses to devote it.

"*Strictures on the use of the term congestive as applied to low forms of fever, with some general observations on the pathology of these diseases.*" *By Isaac Parish, M. D., Philadelphia.* This is an article published in the July, last number of the American Med. Journal. The author objects, and, in our opinion, with propriety, to the use of the term congestive as applied to fevers, deeming the pathology implied by its application hypothetical. Words, in such cases, do much mischief by serving to entrench and perpetuate error. The author might easily extend his criticisms to other, and, indeed, to the greater portion of the common nomenclature of our science.

"*Address delivered at Woodstock, Vermont, at the anniversary of the Vermont Medical College, June 11th,* 1845, *by W. R. Ranney, M. D.*"

"*The third annual Reports of the Superintendent and Trustees of the New Hampshire Asylum for the Insane.*" We perceive by a late No. of the Boston Journal, that Dr. Chandler, the able Superintendent, has resigned his connection with this institution.—The Trustees eulogize in the warmest terms, his ability and fidelity; and the statistics contained in the above reports, exhibit a large per centage of recoveries. We trust that his efforts will not be lost to this very important branch of medical philanthropy.

"*Southern Medical and Surgical Journal, Augusta, Georgia. Edited by Paul F. Eve, M. D., and J. P. Garvin, M. D.*" This is a monthly of 62 pages, presenting a very prepossessing *external* appearance, and in its *internal* merits it will compare very favorably with any of our exchanges.

"*The British American Journal of Medical and Physical Science. Edited by Archibald Hall, M. D., and Robert L. McDonnell, M. D., Montreal, Canada.*" Nos. 7 and 8. This is a monthly of 27 quarto pages. It is not confined strictly to medical subjects, but embraces those of physical science generally. The numbers with which we have been favored, give evidence of talent and usefulness.

"*St. Louis Medical and Surgical Journal for Sept.*"

"*Western Lancet for August.*"

"*New York Medical Intelligencer and Eclectic Gazette.*" Nos. 1 and 2. This is a new Journal, emanating from the metropolis, published bi-weekly, containing 16 quarto pages. It consists of selections from foreign journals exclusively. For those who have not access to foreign journals, this must prove acceptable, since it is designed to select the portions which are of general and peculiar interest, omitting those of less importance, and which are interesting only from local associations.

"*New Orleans Medical and Surgical Journal for September.*" By an arrangement with the editors of this, and another projected periodical, the editorial force has been doubled. The Editors are W. M. Carpenter, M. D., E. D. Fenner, M. D., J. Harrison, M. D., and A. Hester, M. D. This Journal is conducted with spirit and ability.—We commend it to those who desire information on the diseases and modifications of disease peculiar to the South. We have been much interested in the article on Yellow Fever, by Dr. Harrison, one of the editors,

"*Boston Medical and Surgical Journal.*" This periodical is too well known to require any eulogium from us. It has greeted us with weekly promptitude for many years, and that it may continue to do so, constitutes one of our agreeable anticipations for the future.

---

*Vacant Professorship.*—We find the following notice in a number of the Lexington Observer, which has been forwarded to us. We publish it with great pleasure, as we regard this method of filling vacancies in Medical Schools, if carried out by an impartial decision upon the legitimate claims of applicants, worthy of general imitation:

*To the Medical Public.*—The chair of Obstetrics and the Diseases of Women and Children in the Medical Department of Transylvania University, is at present vacant; and with a view to fill it in the best possible manner, applications for the place are invited from the members of the Medical profession. Communications on the subject must be forwarded to the Dean of the Medical Faculty prior to the 30th day of January next, when the appointment will be made. It will be required, in conformity with a resolution of the Board of Trustees, that the person selected shall make Lexington his permanent residence. The name of no one but the successful candidate will be made public.

M. C. JOHNSON, Ch'm. B. T. T. U.

Lexington, Sept. 29, 1845.

